# Prevalence and Risk Factors of Bovine Viral Diarrhea Virus Antibodies in Dairy Herds of Bangladesh

**DOI:** 10.3390/vetsci12080739

**Published:** 2025-08-07

**Authors:** Md. Saifullah Mahmud Sajeeb, Md. Shaffiul Alam, Md. Nazmul Islam, Md. Monirul Islam, Bishwo Jyoti Adhikari, Shanta Islam, Md. Siddiqur Rahman, A. K. M. Anisur Rahman

**Affiliations:** 1 Epidemiology and Preventive Medicine Laboratory, Department of Medicine, Bangladesh Agricultural University, Mymensingh 2202, Bangladesh; saifullah45775@bau.edu.bd (M.S.M.S.); nazmul.1901007@bau.edu.bd (M.N.I.); siddique.medicine@bau.edu.bd (M.S.R.); 2 Faculty of Veterinary Science, Bangladesh Agricultural University, Mymensingh 2202, Bangladesh; joyoti.2001206@bau.edu.bd (B.J.A.); shanta.2001138@bau.edu.bd (S.I.)

**Keywords:** BVD, antibody cELISA, semen source, herd size, physical condition, milk yield, age

## Abstract

A cross-sectional study was conducted in Bangladesh from January 2023 to December 2024 to assess the prevalence of BVDV antibodies and associated risk factors in dairy herds. Initially, 138 bulk milk samples were collected from herds across 14 districts and screened using a commercial ELISA for BVDV antibodies. Subsequently, 767 individual milk samples were tested from cows in herds that screened positive. The overall herd-level seroprevalence was 72.5% (95% CI: 64.1–79.6), while the cow-level seroprevalence was 93.3% (95% CI: 91.3–94.9%). Within-herd seroprevalence varied from 81.8% to 100%. Herds with more than 70 cows had 31.95 times higher odds of BVDV seropositivity than those with 13–23 cows. BVD seroprevalence was significantly higher in the third, fifth, and eighth semen sources compared to the reference source. Cows older than 8 years had 4.53 times higher odds of seropositivity than those aged up to 4 years. Cows in thin physical condition had 13.02 times higher odds of seropositivity than those in normal condition. Conversely, cows producing over 8.8 kg of milk daily had a significantly lower seroprevalence of BVDV compared to those producing less than 8.8 kg, with an odds ratio of 0.41 (95% CI: 0.17–0.98). These findings suggest that BVDV exposure is widespread in dairy herds in Bangladesh. Regular serological screening, the use of semen from BVD-free bulls, and prioritizing surveillance for thin cows and low milk producers are essential. Improving nutrition, herd management, and targeted monitoring may help reduce BVDV transmission and improve herd productivity.

## Simple Summary

We performed a cross-sectional study in Bangladesh from January 2023 to December 2024 to estimate the prevalence of Bovine Viral Diarrhea Virus (BVDV) antibodies and identify associated risk factors in dairy cattle. Bulk milk samples from 138 herds across 14 districts were screened using a commercial antibody ELISA, and we subsequently tested individual milk samples from 767 cows. The results showed that 72.5% of herds and 93.3% of cows tested were seropositive for BVDV antibodies. In some herds, all tested cows were seropositive. Larger herds (>70 cows) were significantly more likely to have higher BVD seroprevalence than smaller herds (13–23 cows). Certain semen sources were also associated with increased seropositivity. Older cows and those in poor physical condition were more likely to be seropositive, while cows producing more than 8.8 kg of milk daily were less likely to have BVDV antibodies. These findings underscore the highly endemic nature of BVDV exposure in Bangladeshi dairy herds. The study suggests the importance of regular serological screening, the use of BVDV-free semen, and targeted monitoring of high-risk animals. Improvements in nutrition and herd management may also contribute to reducing transmission and enhancing productivity.

## 1. Introduction

Bovine viral diarrhea (BVD) is a highly infectious disease that results in substantial economic losses in the global cattle industry [1]. The BVD Virus (BVDV), responsible for BVD, is a single-stranded, positive-sense RNA virus belonging to the Pestivirus genus within the Flaviviridae family [2]. This genus also includes the classical swine fever virus and the border disease virus [2,3]. BVDV is divided into two main species, BVDV-1 and BVDV-2, with a less common proposed third species, BVDV-3 (HoBi-like viruses). Only one study in Bangladesh has characterized BVDV, detecting BVD-3 [4]. BVDV has two biotypes, cytopathogenic (cp) and non-cytopathogenic (ncp), with the ncp biotype being more common. Cattle of all breeds and ages are the natural hosts of BVDV, but other animals like goats, sheep, camels, pigs, and giraffes can also be infected. In immunocompetent animals, acute infection is either asymptomatic or associated with mild, nonspecific signs, such as transient fever, decreased appetite, or mild respiratory signs. The ncp biotype typically causes self-limiting infections within two to three weeks and confers lasting immunity; however, it often leads to transient immunosuppression, making animals susceptible to secondary infections and negatively affecting reproductive performance. More severe BVDV infection outcomes—including stillbirths, abortions, congenital defects, and the birth of persistently infected (PI) calves—are usually associated with in utero transmission of the ncp biotype during the first trimester of gestation. PI calves, which may appear clinically normal, are crucial in maintaining BVDV circulation. In contrast, mucosal disease is a rare but fatal outcome that occurs when a PI animal becomes superinfected with a closely related cp strain of the same virus. This condition is characterized by severe clinical signs, including high fever, gastrointestinal tract erosions, salivation, ulceration, lameness, and profuse diarrhea, often leading to rapid death [5]. There is no published information on BVDV biotype distribution in Bangladesh due to the lack of virus isolation and in vitro characterization studies, as most studies rely on serology, which does not distinguish biotypes.

Bangladesh, one of the world’s most densely populated countries, is home to nearly 24 million cattle, resulting in 145 ruminants per square kilometer. The cattle industry supports approximately 15% of the country’s workforce and contributes about 3% to the agricultural GDP [4]. Bovine Viral Diarrhea is an economically significant disease of cattle worldwide, including in South and Southeast Asia. Several studies from the region report seroprevalence levels ranging from moderate to high, often in the absence of formal control programs. In India, for instance, the seroprevalence varied between 13.2 and 56.7% [6,7]. In Nepal, the seroprevalence varied from 7.8% to 10.9% [8,9]. However, in Myanmar, the prevalence of BVDV antibodies in the bulk tank milk of small-scale dairy herds was 2.10% [10]. Although BVD is known to be present in neighboring countries and could have a significant economic impact on Bangladesh’s large cattle population, research on the seroprevalence of BVD in Bangladesh has been limited [11,12]. The reported prevalence of BVD in Bangladesh was 51.1% in both studies [11,12]. Currently, no national BVDV control program or vaccination strategy exists in Bangladesh. Consequently, diagnosis is rarely pursued, and PI animals remain undetected, contributing to the silent spread of the virus within and between herds.

Bovine Viral Diarrhea is known for its stealthy nature and the presence of PI animals, which act as efficient reservoirs. These factors have contributed to its widespread distribution among cattle populations worldwide [13]. In Bangladesh, previous research was limited to 94 serum samples collected from two districts: Chattogram and Barishal [11,12]. This study aims to estimate the seroprevalence of BVD in dairy cattle and identify the risk factors associated with the disease, covering large geographic regions and using an appropriate sample size for the first time in Bangladesh.

## 2. Materials and Methods

### 2.1. Study Design, Study Population, and Target Population

**Figure 1:**
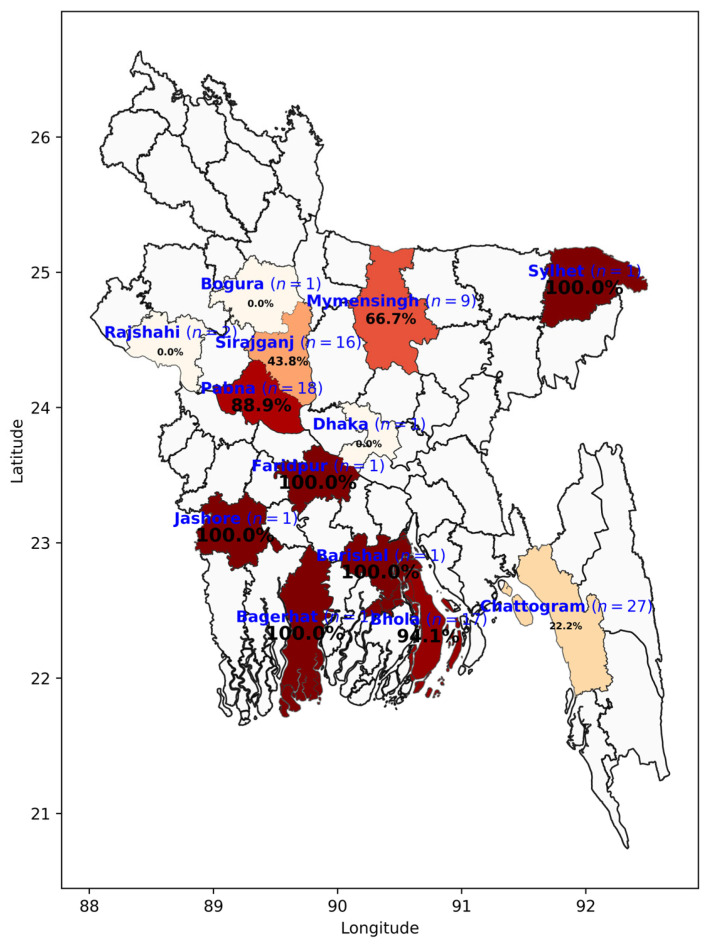
Map of Bangladesh showing the study areas and herd-level seroprevalence of Bovine Viral Diarrhea across districts. The number ‘*n*’ indicates the number of herds tested in each district. The map was created using Python version 3.12.4 in Jupyter Notebook environment (version 7.0.8), utilizing Matplotlib 3.10.5 and Geopandas 1.1.1 libraries. Bangladesh district shape files were obtained from the GADM maps and data (https://gadm.org/ accessed on 25 July 2025).

A cross-sectional study was conducted from January 2023 to December 2024, targeting dairy cattle in Bangladesh. The study population consisted of dairy herds from 14 selected districts across the country (Figure 1). Since vaccination against BVD is not currently practiced in Bangladesh, the sampled animals were assumed to be unvaccinated. Milk samples were collected from dairy cows within the selected herds and all sampling procedures were carried out by registered and well-trained veterinary professionals.

### 2.2. Sampling Size Calculation and Sampling Protocol

The sample size for this study was determined using Equation (1).

(1) $$n=\frac{Z^{2}P(1-P)}{d^{2}}$$

In this equation, *Z* is the Z-score for a 95% confidence level, which is 1.96. *P* represents the expected prevalence, set at 51.1% or 0.511 [12], and *d* is the precision, determined to be 4% or 0.04. Based on these assumptions, the calculated sample size was 600. However, a total of 767 samples were ultimately collected to increase the statistical power of the study and enhance the precision of estimates, particularly for subgroup analyses. Initially, bulk milk samples from 138 conveniently sampled farms were chosen. Then, eight herds were randomly selected from the all the BVD-positive herds until the target sample size of 767 was reached.

### 2.3. Collection of Milk Samples

Approximately 40 mL of composite (bulk) milk from a herd was collected aseptically in a sterile Falcon tube. The same amount of fresh milk was also collected aseptically from each cow in the herd into separate sterile Falcon tubes. Each sample was labeled, immediately frozen, and transported to the Department of Medicine at Bangladesh Agricultural University in an icebox. The samples were stored at −20 °C until further analysis.

### 2.4. Herd- and Cow-Level Data Collection

Herd- and cow-level data on potential risk factors were collected using pretested questionnaires. Herd-level data included herd size; the number of lactating cows, heifers, and calves on the farm; the annual number of abortions; retention of fetal membranes; repeat breeding cases; daily milk yield; and the source of semen for breeding. Cow-level data encompassed age, breed, physical condition, pregnancy status, history of abortion, retained fetal membrane, reproductive disorders, number of calves, lactation stage, and daily average milk yield.

### 2.5. Processing of Milk Samples

The milk samples were thawed at room temperature and left until the cream separated from the lactoserum, with the cream floating on top and the lactoserum at the bottom. The lactoserum was then transferred into 1.5 mL Eppendorf tubes and placed in a 96-well plate (non-ELISA plate). Wells A1 and B1 contained positive controls, while C1 and D1 contained negative controls. The remaining 92 wells were filled with lactoserum to facilitate the rapid transfer of both controls and samples to the ELISA plate using a 12-channel micropipette.

### 2.6. Competitive Enzyme-Linked Immunosorbent Assay (cELISA)

The collected milk samples were analyzed using ID Screen BVD p80 Antibody competitive ELISA kits (BVDC-10P, Innovative Diagnostics, Grabels, France) as per the manufacturer’s instructions to detect BVDV antibodies. Reagents were brought to room temperature (21 °C ± 5 °C) before use. Homogenization was achieved by inversion or vortexing. Then, 100 µL of positive control was added to wells A1 and B1, 100 µL of negative control to wells C1 and D1, and 100 µL of each milk sample to the remaining wells. The plate was covered with sterile plastic and incubated overnight at 5 °C (±3 °C). The wells were then emptied and washed five times with at least 300 µL of wash solution. Ready-to-use conjugate was added to each well and incubated at 21 °C (±5 °C) for 30 min (±3 min). The wells were washed 3 times with at least 300 µL of wash solution. Substrate solution was added to each well and incubated at 21 °C (±5 °C) in the dark for 15 min (±2 min). Stop solution was added to each well in the same order as in other steps to stop the reaction. The Optical Density (OD) value at 450 nm was read using an ELISA reader.

### 2.7. Test Validation and Interpretation

The test was considered valid if the mean value of the negative control OD (ODNC) exceeded 0.7 and if the mean value of the positive control OD (ODPC) was less than 30% of the ODNC. The competition percentage (S/N%) for each sample was calculated using the formula S/N% = (ODsample/ODNC) × 100. A result was classified as positive if the S/N% was 65% or less and negative if it was greater than 65%.

### 2.8. Data Analysis

#### 2.8.1. Descriptive Statistics

Numeric data such as OD value, S/N%, herd size, and the number of lactating cows, heifers, and calves on the farm, along with the number of abortions, retention of fetal membranes, repeat breeding cases per year, daily milk yield at herd level, age, lactation stage, number of calves, lactation stage, and daily average milk yield, were summarized using the ‘summary’ function in R version 4.5.1 (R Foundation for Statistical Computing, Vienna, Austria). The prevalence of BVDV antibodies and its distribution across various categories were calculated using the ‘tabpct’ function from the ‘epiDisplay’ package in R 4.5.1. Additionally, the seroprevalence of BVD at both the herd and cow levels, along with its 95% confidence interval, was determined using the ‘prop.test’ function in R version 4.5.1.

#### 2.8.2. Identification of Risk Factors

##### Herd-Level Risk Factors

A herd was classified as BVDV-seropositive if its bulk milk tested positive for BVDV-specific antibodies using the commercial cELISA test. Continuous variables were converted to categorical variables based on quartiles or median values to facilitate analysis. Initially, univariable logistic regression analyses were performed with bulk-milk-level BVDV seropositivity as the outcome and each independent variable as a predictor. Variables with a *p*-value ≤ 0.20 were selected for inclusion in the multivariable model. Multicollinearity among the selected variables was assessed using the vif() function from the “car” package in R (version 4.5.1). A generalized variance inflation factor (GVIF) > 2 was considered indicative of multicollinearity [14]. Stepwise multivariable logistic regression was used to build the final model. Confounding and interaction terms were evaluated using established methods [15].

##### Cow-Level Risk Factors

A cow was classified as BVDV-seropositive if its milk sample tested positive in the cELISA test. Continuous variables such as age, lactation stage, number of calves, and milk yield were converted to categorical variables, mainly based on quartiles and occasionally on medians to ensure meaningful groupings. This data-driven approach avoids arbitrary cut-off points and reflects the actual distribution in our sample. Quartiles create balanced subgroups and improve association interpretability, especially without established clinical thresholds. To assess individual-level risk factors, a univariable mixed-effects logistic regression was performed for each predictor variable, with BVDV seropositivity as the outcome and farm included as a random intercept to account for clustering within herds. In the univariable screening, any variables associated with BVDV seropositivity with a *p*-value of ≤0.20 were selected for the multivariable model. Multicollinearity among the selected variables was assessed using the same method described in the previous section [14]. A stepwise mixed-effects forward multivariable logistic regression model was used to determine the final model. Confounding and interaction were also checked using similar methods described in the previous section [14]. The data used to identify herd- and cow-level risk factors are provided in Supplementary Data S1 and Data S2, respectively.

## 3. Results

### 3.1. Summary Statistics

This study involved 138 dairy herds across 14 districts in Bangladesh (Figure 1). The mean herd size was 119.0, ranging from 4 to 2850 animals. Among these herds, the mean number of lactating cows was 37 (range: 0 to 1100), while the mean number of heifers was 26 (range: 0 to 698). The mean number of abortions per herd was 3 (range: 0 to 32), and the mean number of retained fetal membrane cases was 4 (range: 0 to 70). The mean number of anestrus cases per herd was 2 (range: 0 to 37), and the mean number of repeat breeding cases was 10 (range: 0 to 600). The daily mean milk yield per herd was 216.12 kg (range: 8 to 6400 kg).

A total of 767 cow milk samples were tested from eight herds in Bangladesh that tested positive for BVDV antibodies. The mean age of the cows was 6.3 years (range: 1.5–17.5 years). Each cow had a mean of 3 calves (range: 0–10). The mean lactation stage was 5.5 months (range: 1–11 months), and the mean body weight was 346.3 kg (range: 130–800 kg). The daily mean milk yield was 8.81 kg (range:1–23 kg).

### 3.2. Herd- and Cow-Level BVD Seroprevalence

**Figure 2:**
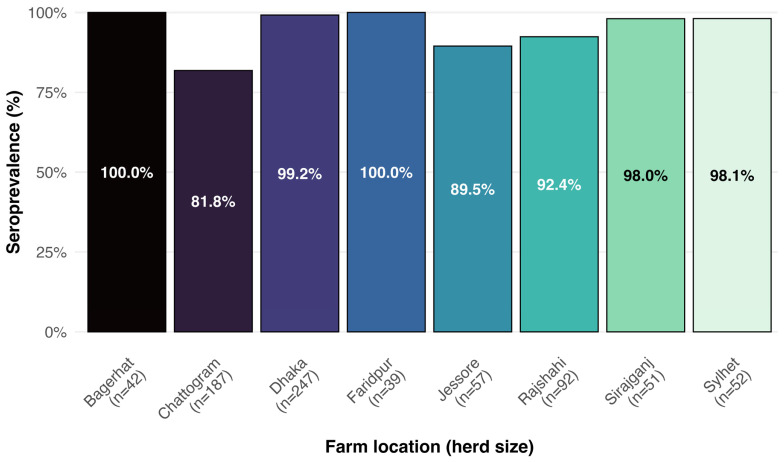
Prevalence of BVDV antibodies in cows from herds with positive serology.

The overall herd-level BVD seroprevalence was 72.5% (95% confidence interval [CI]: 64.1–79.6) based on antibody detection in bulk milk samples. Among the herds that tested positive for antibodies, the overall cow-level seroprevalence was 93.3% (95% CI: 91.3–94.9%). Within these seropositive herds, the within-herd seroprevalence varied from 81.8% to 100% (Figure 2).

### 3.3. Risk Factors

#### 3.3.1. Herd

In the univariable screening, herd size, the number of lactating cows, and semen source were associated with BVDV seropositivity at a *p*-value of ≤0.20 and were therefore included in the multivariable logistic regression model (Supplementary Table S1). No evidence of multicollinearity was found among these independent variables. In the final multivariable logistic regression model, herd size and semen source were identified as significant herd-level risk factors for BVDV seropositivity. Herds with more than 70 cows had 31.95 times higher odds of being BVDV-seropositive (95% CI: 4.9–208.43) compared to herds with 13 to 23 cows. Compared to the reference semen source, the odds of BVDV seroprevalence were significantly higher in the third (odds ratio (OR): 24.47, 95% CI: 4.09–146.53), fifth (OR: 8.99, 95% CI: 2.23–36.32), and eighth (OR: 23.55, 95% CI: 3.8–146.04) semen sources (Table 1).

A total of 138 herds from 14 different districts across Bangladesh were examined. Herds in three of these districts tested negative for BVDV antibodies. In the remaining districts where BVDV seropositivity was detected, the herd-level seroprevalence varied from 22.2% to 100% (Figure 2).

#### 3.3.2. Cow

In the univariable screening, age, breed, physical condition, number of calves, and milk yield were associated BVDV seropositivity at a *p*-value ≤ 0.20 (Supplementary Table S2).

No multicollinearity was found among these explanatory variables. In the final mixed-effects multivariable logistic regression model, age, physical condition, and milk yield were identified as significant cow-level risk factors for BVDV seropositivity (Table 2). Cows older than 8 years had 4.53 times higher odds of being seropositive for BVDV (95% CI: 1.90–10.77) compared to those aged up to 4 years. Cows in thin physical condition had 13.02 times higher odds of seropositivity (95% CI: 1.67–10.77) than those in normal physical condition. Furthermore, cows producing more than 8.8 kg of milk daily had significantly lower seropositivity of BVDV (OR: 0.41; 95% CI: 0.17–0.98) compared to those producing ≤ 8.8 kg of milk daily (Table 2).

## 4. Discussion

This study provides the first comprehensive description of BVD seroprevalence and associated risk factors across a large geographic area in Bangladesh. The findings demonstrate that BVDV seropositivity is highly endemic at both herd and individual levels in Bangladesh. This emphasizes the importance of enhanced biosecurity, especially in large dairy operations, and the necessity of rigorous breeding bull screening to prevent further spread. Improving nutrition, herd management, and targeted monitoring may help reduce BVDV transmission and improve herd productivity.

The herd-level seroprevalence of BVD was notably high, indicating a significant presence of the disease within the studied population. Nearly three-quarters of the herds were affected, suggesting widespread exposure or transmission within the region. No previous studies in Bangladesh have reported the herd-level seroprevalence of BVD, making it difficult to compare any results with the findings of this study. The within-herd seroprevalence among positive herds was also very high, ranging from 81.8% to 100%. It is reported that about 70–90% of BVD infections are subclinical, as observed in this study [16]. The comparatively lower prevalence of BVD in neighboring countries [6,7,8,9,10] may be attributed to several region-specific factors. In some of these countries, veterinary service delivery systems are more structured and benefit from more consistent implementation of farm-level disease surveillance and animal health programs. Additionally, the use of artificial insemination (AI) through organized government or private breeding programs may reduce the risk of BVD transmission via tested breeding bulls—a recognized route of disease spread. In contrast, dairy farming in many parts of Bangladesh remains largely informal, with limited veterinary oversight, and a heavy reliance on untested breeding bulls. Moreover, awareness and implementation of biosecurity practices among Bangladeshi dairy farmers are generally poor [17]. Vaccination against BVD is not yet practiced in Bangladesh. These factors likely contribute to the higher BVD seroprevalence observed in our study region compared to neighboring countries. 

Herds with more than 70 cows showed significantly higher BVD seroprevalence compared to herds with 13 to 23 cows. A similar finding has also been reported from Ethiopia [18]. Larger herds often result in increased animal-to-animal contact, which accelerates the transmission of infectious diseases like BVD. The higher density and frequency of interactions among cows create an environment conducive to virus spread. Additionally, managing biosecurity measures effectively becomes more difficult as herd size increases, increasing the risk of disease outbreaks due to potential lapses. Larger herds also tend to introduce new animals more frequently, which can serve as a source of BVDV if quarantine and health-screening protocols are not strictly enforced. While quarantine is a key biosecurity measure, it is insufficient on its own to prevent the introduction of BVDV if a PI animal is purchased. PI animals remain infected and infectious for life, continuously shedding the virus regardless of their clinical appearance. Consequently, they can introduce and sustain BVDV within a herd even when standard quarantine procedures are in place. Therefore, in addition to quarantine, pre-purchase testing—particularly antigen- or PCR-based diagnostics—is essential to detect and exclude PI animals before herd entry. Strengthening biosecurity protocols with a specific focus on identifying and preventing the introduction of PI animals is crucial for effective BVDV control and herd health protection.

The current study identified a significant association between semen source and herd-level BVDV seropositivity. Three semen sources showed a significantly higher seroprevalence of BVD than the reference semen source. BVD is known to be a semen-borne disease [19]. As a result, the Animal Diseases Rule of Bangladesh [20] recommends that all breeding bulls be free from six semen-borne diseases: brucellosis, tuberculosis, BVD, infectious bovine rhinotracheitis, campylobacteriosis, and trichomoniasis. However, breeding bulls in both government and private bull stations are currently only tested for brucellosis and tuberculosis. Although we did not directly assess semen quality or test bulls for BVDV, the observed pattern supports a plausible hypothesis that frozen semen may serve as a significant transmission route in Bangladesh. Further investigation into bull health status and semen testing practices is warranted.

The seroprevalence of BVD was significantly higher in cows over eight years old compared to those aged four years or younger. This finding is consistent with results from other studies [18,21,22]. The higher seroprevalence in older cows may be attributed to their longer lifespan, which increases the likelihood of cumulative exposure to BVDV over time. It is important to note that PI animals are unlikely to survive to older ages; therefore, the higher seroprevalence in older animals reflects past exposure rather than persistent infection.

Cows in a thin physical condition had a significantly higher seroprevalence of BVD compared to those in a normal physical condition. Similar findings have been reported by other authors [12,23]. It is challenging to establish a causal relationship from a cross-sectional study. However, poor body condition may be associated with a compromised immune system, making these cows more vulnerable to BVD infection. The weakened immune defenses in undernourished or stressed animals could allow the virus to enter and replicate more easily, resulting in a higher incidence of the disease.

Our study found a negative correlation between BVDV seropositivity and milk yield, indicating that cows producing less milk (up to 8 kg) have a significantly higher prevalence of BVDV antibodies compared to those producing more milk (>8 kg). Although this cross-sectional study cannot establish a causal relationship between BVDV seropositivity and milk yield, it is well-documented that BVD-infected cows produce less milk than non-infected ones. This suggests a consistent pattern, indicating that BVD negatively affects milk production [24].

## 5. Conclusions

Bovine Viral Diarrhea Virus seropositivity is highly endemic in both herds and animals across Bangladesh, indicating extensive historical exposure. This highlights the urgent need to strengthen biosecurity measures, particularly in large dairy operations where transmission risks are higher. Screening breeding bulls for BVDV is essential because semen plays a significant role in viral dissemination. Effective BVDV control requires an integrated approach, including improved nutrition, herd management, targeted surveillance of high-risk animals, and the implementation of tailored vaccination programs. Farmer-focused extension services are also crucial to promote awareness and adoption of best practices in biosecurity and reproductive health. Collectively, these measures can help reduce BVDV transmission, improve herd productivity, and mitigate the economic impact on Bangladesh’s dairy sector. Future studies should aim to characterize BVDV species diversity and estimate the prevalence of PI animals to support the development of effective control and eradication strategies.

## Figures and Tables

**Table 1:** Risk factors for herd-level BVDV seropositivity identified in the final multivariable logistic regression model.

Variable	Categories	Estimate (SE)	Odds Ratio (95% CI)	*p*-Value
Herd category				
	≤13	0.56 (0.70)	1.27 (0.33–4.93)	0.42
	>13 to 23	-	-	-
	>23 to 70	0.36 (0.67)	1.94 (0.49–7.65)	0.59
	>70	2.61 (0.76)	31.95 (4.9–208.43)	<0.001
Semen source				
	Source 1	Not analyzed	Not analyzed	Not analyzed
	Source 2	1.01 (0.93)	2.75 (0.44−17.11)	0.28
	Source 3	3.19 (0.91)	24.47 (4.09−146.53)	<0.001
	Source 4	Not analyzed	Not analyzed	Not analyzed
	Source 5	2.19 (0.71)	8.99 (2.23−36.32)	0.002
	Source 6	Reference	Reference	Reference
	Source 7	Not analyzed	Not analyzed	Not analyzed
	Source 8	3.16 (0.93)	23.55 (3.8−146.04)	<0.001

**Table 2:** Risk factors for cow-level BVDV seropositivity identified in the final mixed-effects multivariable logistic regression model.

Variables	Category	Estimate	SE	Odds Ratio (95% CI)	*p*-Value
Age (years)					
	Up to 4	−0.13	0.56	0.87 (0.28–2.67)	0.81
	4 to 5.6	Reference	–	–	–
	5.6 to 8	0.64	0.46	1.89 (0.75–4.76)	0.17
	>8	1.51	0.44	4.53 (1.90–10.77)	<0.001
Physical condition					
	Thin	2.56	1.04	13.02 (1.67–101.82)	0.01
	Normal	Reference	–	–	–
Milk yield (Kg)					
	≤8.8	Reference	–	–	–
	>8.8	−0.88	0.44	0.41 (0.17–0.98)	0.04

## Data Availability

The data supporting reported results are provided as Supplementary Materials.

## Supplementary Materials

The following supporting information can be downloaded at https://www.mdpi.com/article/10.3390/vetsci12080739/s1. The herd and cow-level data used in this study, along with the univariable screening results for both herd and cow levels, are available as Data S1: Herd level BVD; Data S2: Cow level BVD; Table S1: Univariable association between herd level BVD status and other explanatory variables; Table S2: Results of the univariable association between cow-level BVD status and other explanatory variables.

## Abbreviations

The following abbreviations are used in this manuscript:

LDDP	Livestock and Dairy Development Project
ELISA	Enzyme-Linked Immunosorbent Assay
BVD	Bovine Viral Diarrhea
CI	Confidence Interval
OD	Optical Density
S/N	Sample to Negative Ratio
OR	Odds ratio
VIF	Variance Inflation Factor
cELISA	Competitive Enzyme-Linked Immunosorbent Assay
