# Supplementary material for: Prevalence and Risk Factors of Bovine Viral Diarrhea Virus Antibodies in Dairy Herds of Bangladesh
Source: Vet Sci. 2025 Aug 7;12(8):739. doi: 10.3390/vetsci12080739 (PMC12389986; doi:10.3390/vetsci12080739)
Supplement: Supplementary file 1 [file vetsci-12-00739-s001.zip › Table S1 Univariable association between herd level BVD status and other explanatory variables.pdf]

Table S1. Univariable association between herd level BVD status and other explanatory variables.

Variables	Category	Tested	Positive (%)	OR (95% CI)	*P*-value
**Herd size**					0.05
	≤ 13	37	31 (83.8)	4.02 (1.31–12.3)	
	> 13–24	32	18 (56.2)	Reference	
	> 24–70	35	24 (68.6)	1.70 (0.63–4.61)	
	> 70	34	27 (79.4)	3.00 (1.01–8.89)	
**Lactating cow number**					0.12
	≤ 5	35	29 (82.9)	3.45 (1.15–10.38)	
	> 5–12	36	21 (58.3)	Reference	
	> 12–31	32	23 (71.9)	1.83 (0.66–5.04)	
	> 31	35	27 (77.1)	2.41 (0.86–6.75)	
**Number of calves**					0.39
	≤ 4	79	55 (69.6)	Reference	
	> 4	59	45 (76.3)	1.4 (0.65–3.02)	
**Abortion case**					0.39
	≤ 4	79	55 (69.6)	Reference	
	> 4	59	45 (76.3)	1.4 (0.65–3.02)	
**Milk yield (Kg)**					0.53
	≤ 110	75	56 (74.7)	1.27 (0.60–2.69)	
	> 110	63	44 (69.8)	Reference	
**Retention of Placenta**					0.29
	≤ 2	79	60 (75.9)	1.5 (0.71–3.18)	
	> 2	59	40 (67.8)	Reference	
**Repeat Breeding case**					0.25
	≤ 2	69	47 (68.1)	Reference	
	> 2	69	53 (76.8)	1.55 (0.73–3.30)	
**Semen source**					0.008
	Source 1	1	0 (0)	Not analyzed	
	Source 2	8	5 (62.5)	1.13 (0.24–5.3)	
	Source 3	23	21 (91.3)	7.12 (1.49–34.01)	
	Source 4	4	4 (100)	Not analyzed	
	Source 5	28	22 (78.6)	2.49 (0.85–7.29)	
	Source 6	47	28 (59.6)	Reference	
	Source 7	5	0 (0)	Not analyzed	
	Source 8	22	20 (90.9)	6.79 (1.42–32.48)	
**Overall**		138	100 (72.5)		

OR: Odds Ratio, CI: Confidence Interval
