# Supplementary material for: Prevalence and Risk Factors of Bovine Viral Diarrhea Virus Antibodies in Dairy Herds of Bangladesh
Source: Vet Sci. 2025 Aug 7;12(8):739. doi: 10.3390/vetsci12080739 (PMC12389986; doi:10.3390/vetsci12080739)
Supplement: Supplementary file 1 [file vetsci-12-00739-s001.zip › Table S2 Results of the univariable association between cow-level BVD status and other explanatory variables.pdf]

Table S2. Results of the univariable association between cow-level BVD status and other explanatory variables.

Variables	Category	Tested	Positive (%)	OR (95% CI)	*P*-value
**Age (years)**					0.06
	Up to 4	211	200 (94.8)	1.37 (0.51 – 3.69)	
	4 to 5.6	178	161 (90.4)	Reference	
	5.6 to 8	194	184 (94.8)	2.37 (1.01 – 5.58)	
	> 8	184	171 (92.9)	2.80 (1.25–6.28)	
**Breed**					0.014
	Indigenous	118	117 (99.2)	12.78 (1.68–97.15)	
	Cross	649	599 (92.3)	Reference	
**Physical condition**					0.007
	Normal	659	609 (92.4)	Reference	
	Thin	108	107 (99.1)	15.19 (2.10–109.92)	
**Pregnancy**					0.94
	No	532	500 (94.0)	1.02 (0.54–1.93)	
	Yes	235	216 (91.9)	Reference	
**Reproductive Disorders**					0.65
	Anestrus	110	93 (84.5)	Reference	
	Repeat Breeding	252	245 (97.2)	1.53 (0.56–4.17)	
	No	405	378 (93.3)	1.04 (0.49–2.18)	
**Abortion**					0.86
	No	739	688 (93.1)	Reference	
	Yes	28	28 (100)	185201500 (0–Inf)	
**Retention of Placenta**					0.71
	No	698	651 (93.3)	Reference	
	Yes	69	65 (94.2)	1.23 (0.41–3.68)	
**Number of Calves**					0.08
	≤ 2	448	418 (93.3)	Reference	
	> 2	319	298 (93.4)	1.74 (0.93–3.27)	
**Lactation Stage (months)**					0.46
	Up to 3	226	203 (89.8)	Reference	
	3 to 5	163	155 (95.1)	1.05 (0.44–2.53)	
	5 to 8	248	236 (95.2)	1.53 (0.71–3.32)	
	> 8	130	122 (93.8)	0.72 (0.26–1.97)	
**Milk Yield (Kg)**					0.007
	≤ 8.8	425	377 (97.7)	0.34 (0.15–0.74)	

	> 8.8	342	335 (88.9)	Reference	
**Mastitis**					0.29
	No	674	627 (93.0)	Reference	
	Yes	93	89 (95.7)	1.85 (0.59–5.80)	
**Overall prevalence**		767	716 (93.4)		

OR: Odds Ratio, CI: Confidence Interval
